# Deep Phenotyping of Pathology‐Confirmed Benign Lesions in PTEN Hamartoma Tumor Syndrome Patients

**DOI:** 10.1111/cge.14759

**Published:** 2025-04-28

**Authors:** Ane J. Schei‐Andersen, Janneke H. M. Schuurs‐Hoeijmakers, Rachel van der Post, Arjen R. Mensenkamp, Jolanda Schieving, PTEN Study Group, Muriel A. Adank, Mirjam de Jong, Marjolijn C.J. Jongemans, Liselotte P. van Hest, Yvette van Ierland, Edward M. Leter, Maartje Nielsen, Janet R. Vos, Nicoline Hoogerbrugge

**Affiliations:** ^1^ Department of Human Genetics Radboud University Medical Center Nijmegen the Netherlands; ^2^ Radboud Institute for Medical Innovation Radboud University Medical Center Nijmegen the Netherlands; ^3^ Department of Pathology Radboud University Medical Center Nijmegen the Netherlands; ^4^ Department of Pediatric Neurology Radboud University Medical Center Nijmegen the Netherlands

**Keywords:** hamartoma, pathology, phenotype, PHTS, PTEN

## Abstract

PTEN Hamartoma Tumor Syndrome (PHTS) is a rare hereditary syndrome. PHTS has a variable phenotype characterized by benign lesions and increased cancer risks. Clarifying the extent of the benign phenotype could facilitate early recognition of PHTS patients before cancer development. Therefore, we assessed the spectrum, frequency, and age of excerpt of pathology‐confirmed benign lesions in PHTS patients. Pathology reports were collected for 379 patients with a pathogenic *PTEN* variant from the Dutch nationwide pathology databank (Palga). Benign lesions were classified as PHTS‐related based on current clinical diagnostic guidelines and non‐PHTS‐related lesions by ICD‐10 codes. Age of last follow‐up was year of pathology request minus birth year. Analyses were stratified by sex and index status. Patients presented mainly with gastrointestinal (54%), skin (47%), thyroid (31%) and vascular lesions (26%). Males developed lipomas at an early age (9 years (5–34)). Females developed endometrial hyperplasia (16%) at an earlier age (41 years (35–49)) and uterine polyps (13%) more often than the general population. No significant differences were observed between sexes or index and non‐index patients. The results are reflective of current diagnostic guidelines. Early‐onset lipomas may be useful for early detection of PHTS patients. Additionally, uterine polyps should be considered for inclusion as a PHTS‐related lesion.

## Graphical abstract

Gastrointestinal, skin, thyroid, and vascular lesions are frequently present in PTEN Hamartoma Tumor Syndrome patients with little to no difference between sexes or by index status. Early‐onset lipomas (< 20 years) may be useful for early recognition of patients, and uterine polyps should be considered for inclusion as a PHTS‐related lesion.

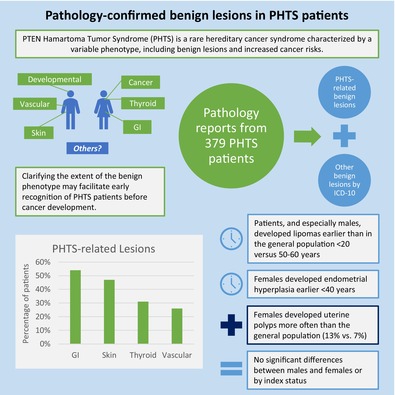

## 1 Introduction

PTEN Hamartoma Tumor Syndrome (PHTS) is a rare hereditary syndrome caused by pathogenic germline variants in the phosphatase and tensin homolog (*PTEN*) gene. Estimates suggest PHTS might be as rare as 1:200 000 worldwide, although this likely is an underestimation [1, 2]. Current PHTS diagnostic guidelines consist of a complex system with multiple major and minor clinical criteria. The guidelines are based on expert medical opinions of the observed phenotype which is characterized by multi‐systemic overgrowth, including malignancies, benign lesions, developmental delay, and autism [1, 2, 3, 4, 5, 6, 7]. The benign lesions associated with PHTS are most often hamartomatous, as the syndrome name denotes. Hamartomas are benign tumor‐like lesions composed of disorganized growth of mature mesenchymal or epithelial tissues indigenous to the organ involved [8]. PHTS patients also have an increased cumulative lifetime risk by age 60 of developing female breast cancer (54%–67%), endometrial cancer (12%–40%) and thyroid cancer (5%–15%) with slight increases for colorectal cancer (0.5%–10%), renal cancer (1%–11%) and melanoma (2%–12%) [1, 5, 9]. As a result, PHTS patients are offered cancer surveillance and/or preventive treatment for their highest cancer risks [2, 6].

Of the observed benign lesions in PHTS, gastrointestinal hamartomatous polyps, Lhermitte‐Duclos disease (dysplastic gangliocytoma), macrocephaly (head circumference > 97th percentile), penile freckling, and mucocutaneous lesions such as trichilemmomas, acral keratosis, neuromas, and papillomas are considered major criteria in the Dutch PHTS guideline [6]. Other benign minor criteria include autism spectrum disorder, glycogenic acanthosis, lipomas, intellectual disability (IQ < 75), testicular lipomatosis, benign thyroid lesions, vascular malformations, and benign breast lesions [6]. In order to receive a clinical PHTS diagnosis, a patient needs to have three major criteria, of which one of the criteria is macrocephaly, Lhermitte‐Duclos disease, or gastrointestinal hamartomatous polyps; or any two major criteria and three minor criteria [3, 6].

The most frequently reported benign lesions in the literature are mucocutaneous lesions, which are estimated to affect almost 100% of PHTS patients [1]. Macrocephaly is also frequently observed and was present in 93% and 83% of PHTS patients in the studies from Bubien et al. and Drissen et al., respectively [2, 10]. A variety of gastrointestinal lesions, including adenomas and hamartomatous polyps, have been observed in over 90% of patients [1, 10]. Benign thyroid disease is estimated to affect 30%–68% of patients, and multinodular goiter was observed in 84% of PHTS patients in Drissen et al.'s study [1, 2, 10]. Vascular malformations have been observed in up to 35%, consisting of arteriovenous malformations, hemangiomas, and lymphangiomas [1, 5, 10]. Neurological disorders, including developmental delay, intellectual disability, and autism spectrum disorder, are estimated to affect 10%–20% of PHTS patients [1, 5].

Sex‐specific benign lesions in the current guideline include penile freckling and testicular lipomatosis, which are both relatively rare in the general population [1, 5, 6, 11]. Fibrocystic breast disease, fibroadenomas, intraductal papillomas, and hamartomas have been observed in up to 47% of female PHTS patients, along with ovarian cysts (37%) and uterine fibromas (25%) [2, 10, 12]. The specificity of female‐specific lesions as part of PHTS is uncertain, as these lesions frequently occur in the general population [1].

Previous studies detailing benign lesions in PHTS were often based on case reports, self‐reported questionnaires, or smaller cohorts. As benign lesions are often not in need of treatment, they are not always removed or studied in detail. As a result, the age of onset and frequency of these lesions are not clear. Some benign lesions also commonly occur in the general population, which makes their clinical value in PHTS uncertain [1]. Detailed knowledge of the benign phenotype could facilitate early recognition of PHTS patients, as some of these lesions may present before cancer development. This would provide clinicians the opportunity to provide preventative treatment and cancer surveillance before cancer development. Furthermore, it provides clinicians insight to determine whether a lesion should be considered part of a PHTS diagnosis.

Therefore, the aim of this study is to investigate the spectrum, frequencies, and age at excerpt of pathology‐confirmed benign lesions in PHTS patients and assess whether recognition can be improved by clinical criteria based on pathology‐confirmed benign lesions in PHTS.

## 2 Methods

### 2.1 Cohort Selection

**FIGURE 1:**
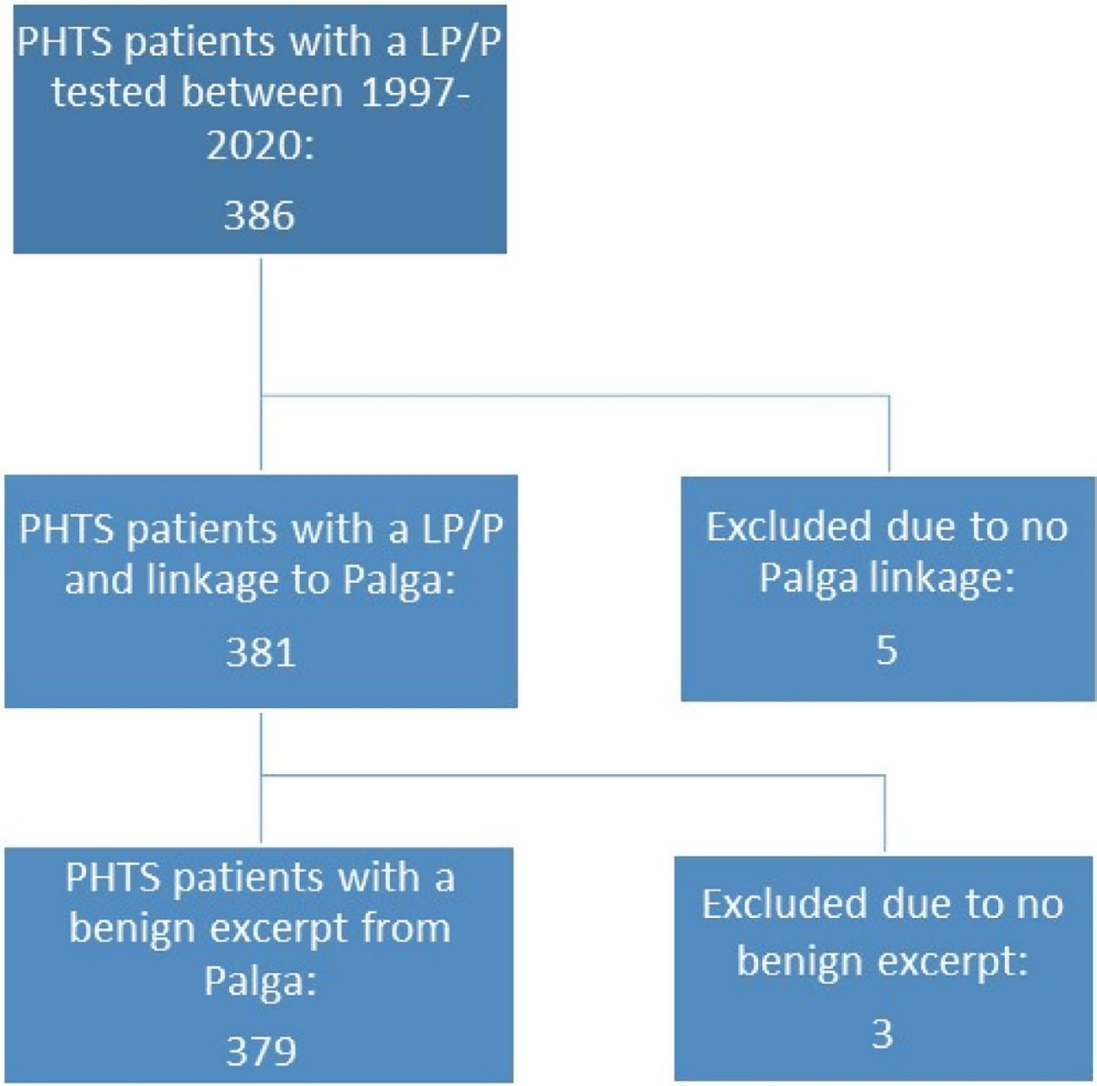
Cohort selection overview. Cohort selection of included patients. DNA testing was performed in the genome diagnostics laboratory of the Radboudumc between 1997 and 2020. 379 (98%) of 386 patients were included in the study. Excluded patients were excluded based on pathology database linkage and/or pathology report availability. Excerpt: Pathology excerpt (description of findings) of tissue, either through biopsy or resection; LP/P: Likely pathogenic/pathogenic; Palga: The Dutch nationwide pathology databank; PHTS: PTEN Hamartoma Tumor Syndrome.

This retrospective cohort study consisted of 379 patients tested for *PTEN* between 1997 and 2020 with a confirmed likely pathogenic/pathogenic (LP/P) *PTEN* germline variant. Patients were tested either due to personal medical history or cascade testing of the familial pathogenic *PTEN* variant. Variants were classified according to ACMG guidelines or the ClinGen PTEN Variant Interpretation Guidelines for PTEN, dependent on the time of diagnosis. The genetic testing results were collected from the diagnostic laboratory (386 patients with a LP/P variant in PTEN) and pathology reports were collected from the Dutch nationwide pathology databank (Palga) (381/386 patients). Patients with no testing result, a variant of uncertain significance (VUS), pathogenic variants not in *PTEN*, or no available pathology reports containing benign lesions (3 patients) were excluded. In total, 379 of 386 patients (98%) with a LP/P PTEN variant were included. An overview of the cohort selection is presented in Figure 1.

The pathology‐confirmed benign lesions were divided into PHTS‐related lesions based on the current Dutch PHTS diagnostic guideline (Table 1) and other observed lesions. The other observed lesions were classified by ICD‐10 codes based on the available information in the pathology reports. The age of the excerpt was based on the creation date of the abstract from the pathology report from which we calculated the patients' age. This was used in place of the age of diagnosis as there may be discrepancies between initial patient contact and pathological diagnosis. Patients were stratified by sex and index status. The last year of follow‐up was defined as the year of the pathology report request (2020). Age at last follow‐up was defined as the year of pathology report request minus year of birth. Selection bias was addressed by separating index cases (i.e., first person in a family to undergo genetic testing) and non‐index cases.

**TABLE 1:** PHTS clinical criteria of the Dutch diagnostic guideline.

Major
Breast cancer
Endometrial cancer
Follicular thyroid cancer
Gastrointestinal hamartomas > 3
Lhermitte‐Duclos disease
Macrocephaly > 97th percentile
Penile freckling
Multiple mucocutaneous lesions: trichilemmomas, acral keratosis, neuromas, oral papillomas
Minor
Autism
Colorectal cancer
Glycogenic acanthosis
Lipomas > 3
Intellectual disability (IQ < 75)
Renal cancer
Testicular lipomatosis
Papillary thyroid cancer
Benign thyroid lesions (adenomas, goiter, etc.)
Vascular malformations
Benign breast lesions (multiple fibroadenomas, intraductal papillomas, etc.)

### 2.2 Statistical Analysis

Data was analyzed in R Studio. Differences in continuous data were assessed by the Wilcoxon rank‐sum test and categorical data by Chi‐Squared or Fisher's exact test where appropriate. The Kruskal–Wallis test was used to assess frequencies stratified by sex and index status. A Bonferroni corrected *p*‐value of 0.0002 was considered statistically significant.

## 3 Results

### 3.1 Patient Characteristics

Of the 379 included PHTS patients, 55% were female and 45% were male. The median age at last follow‐up overall was 38 years (IQR 18–53), 42 years (IQR 23–56) for females and 25 years (IQR 14–49) for males (*p* < 0.0002). Approximately half (56%) of the patients were index cases, 29% were female and 27% were male. Males were significantly younger at genetic testing compared to females (14 years (IQR 5–40) vs. 34 years (IQR 15–45), *p* < 0.0002) although testing was performed in childhood and in adulthood in both sexes. Index cases and index males were significantly younger at genetic testing as well as last follow‐up compared to non‐index cases and index females (*p* < 0.0002) (Table 2).

**TABLE 2:** Patient characteristics.

Characteristics	Total	Female	Male	*p*
Patients, *N* (%)	379 (100%)	210 (55.4%)	169 (44.6%)	1
Age genetic testing, median (IQR)	30 (6–43)	34 (15–45)	14 (5–40)	**< 0.0002**
Age at last follow‐up, median (IQR)	38 (18–53)	42 (23–56)	25 (14–49)	**< 0.0002**
Index case, *N* (%)	213 (56.2%)	112 (29.6%)	101 (26.7%)	0.2502
Index age genetic testing, median (IQR)	15 (4–41)	34 (10–45)	6 (3–26)	**< 0.0002**
Index age at last follow‐up, median (IQR)	26 (14–52)	44 (19–56)	18 (12–39)	**< 0.0002**

*Note:* Bold values indicate significant *P*‐value after bonferroni correction.

### 3.2 PHTS‐Related Benign Lesions

The most frequently observed PHTS‐related benign lesions were gastrointestinal lesions (54%) followed by skin (47%), thyroid (31%) and vascular lesions (26%). Approximately 57% of females developed benign breast lesions. Frequencies of PHTS‐related lesions are presented in Table 3 and Supporting Information Table 1. The frequencies of PHTS‐related lesions in this cohort, PHTS patients from literature, and the general population are presented in Table 4. There were no significant statistical differences observed between sexes or by index status in any of the PHTS‐related lesions.

**TABLE 3:** Frequency of patients per benign lesion and excerpt age.

Feature, *N* (%)	Total	Age, median (IQR)	Female	Female age, median (IQR)	Male	Male age, median (IQR)	*p*, sexes	*p*, age
Gastrointestinal	202 (54%)		128 (61%)		74 (44%)			
Patients above surveillance age > 40 with a GI lesion/total > 40 (%)	143/178 (80%)		96/113 (85%)		47/65 (72%)			
Hamartomatous polyps	96 (25%)		60 (29%)		36 (21%)		1	0.3174
Hamartomatous polyps NOS	33 (9%)	43 (41–47)	19 (9%)	41 (34–47)	14 (8%)	44 (42–47)	1	0.1374
Ganglioneuromas	34 (9%)	43 (39–50)	20 (10%)	41 (36–44)	14 (8%)	44 (41–62)	0.51	0.1387
Lymphoid polyps	1 (0%)	46	1 (0%)	46	0 (0%)	—	—	
Inflammatory and juvenile	28 (7%)	38 (26–44)	20 (10%)	41 (32–50)	8 (5%)	42 (33–47)	0.617	0.9797
Adenomas	55 (15%)	46 (40–58)	37 (18%)	46 (38–56)	18 (11%)	47 (42–63)	1	0.2395
Hyperplastic polyps	35 (9%)	48 (45–56)	24 (11%)	48 (45–53)	11 (7%)	47 (44–61)	0.516	0.6057
Sessile serrated lesions	10 (3%)	49 (46–57)	6 (3%)	53 (49–57)	4 (2%)	46 (43–51)	1	0.2571
Glycogenic acanthosis	6 (2%)	42 (35–52)	1 (0%)	18	5 (3%)	44 (40–54)	0.11	0.3333
Skin	180 (47%)		116 (55%)		64 (38%)			
Lipoma	57 (15%)	24 (10–41)	33 (16%)	30 (22–41)	24 (14%)	9 (5–34)	0.51	0.006589
Trichilemmoma	12 (3%)	35 (30–37)	9 (4%)	32 (28–36)	3 (2%)	38 (38–49)	1	0.01567
Papilloma	25 (7%)	30 (24–38)	16 (8%)	31 (27–39)	9 (5%)	26 (10–38)	0.41	0.4611
Acanthosis nigricans	1 (0.2%)	11 (11–11)	1 (0.4%)	11 (11–11)	0 (0%)	—		
Fibroma	74 (20%)	33 (22–42)	49 (23%)	36 (27–42)	25 (15%)	26 (13–34)	0.038	0.00913
Skin tag	11 (2%)	30 (19–37)	8 (4%)	29 (20–36)	3 (2%)	30 (23–46)	0.36	0.9212
Thyroid	118 (31%)		85 (40%)		33 (20%)			
Patients above surveillance age > 11 with a thyroid lesion/total > 11 (%)	118/341 (35%)		85/201 (42%)		33/140 (24%)			
Benign multinodular hyperplasia	75 (20%)	38 (23–49)	55 (26%)	36 (23–48)	20 (12%)	41 (24–49)	0.9746	0.4831
Follicular adenomas	38 (10%)	35 (23–44)	26 (12%)	33 (22–42)	12 (7%)	39 (32–47)	1	0.2323
Hashimoto's thyroiditis	5 (1%)	41 (40–42)	4 (2%)	42 (41–44)	1 (1%)	16 (16–16)	1	0.4
Other								
Vascular malformation	99 (26%)	27 (20–35)	72 (34%)	27 (21–33)	27 (16%)	26 (16–42)	1	0.9743
Lhermitte‐Duclos disease	8 (2%)	36 (34–41)	6 (3%)	35 (33–42)	2 (1%)	38 (37–39)	0.31	0.6429
Buccal hamartoma	1 (0.2%)	20	1 (0.4%)	20	0 (0%)	—		
Any other abnormality	121 (32%)	33 (22–42)	92 (44%)	35 (23–44)	29 (17%)	26 (19–37)	1	0.1309
Breast	119 (31%)		119 (57%)		0 (0%)			
Patients above surveillance age > 25 with a benign breast lesion/total > 25 (%)	118/240 (49%)		118/155 (76%)		n/a			
Fibrocystic breast disease	66 (17%)	38 (32–45)	66 (31%)	38 (32–45)	0 (0%)	—		
Fibromas/fibroadenomas	40 (11%)	28 (19–39)	40 (19%)	28 (19–39)	0 (0%)	—		
Papilloma	13 (3%)	27 (20–39)	13 (6%)	27 (20–39)	0 (0%)	—		
Uterus	49 (13%)		49 (23%)		n/a			
Patients above surveillance age > 30 with a uterine lesion/total > 30 (%)	49/219 (22%)		49/142 (35%)		n/a			
Fibroid	15 (4%)	46 (38–51)	15 (7%)	46 (38–51)	n/a	—		
Hyperplasia	34 (9%)	41 (35–49)	34 (16%)	41 (35–49)	n/a	—		

**TABLE 4:** Overview of the frequency of benign lesions in the current cohort, PHTS patients from literature and the general population.

Feature, *N* (%)	This cohort (*N*_total_ = 379)	PHTS from literature	General population
Gastrointestinal	202 (54%)	16%–95%	
Hamartomatous polyps	96 (25%)		
Hamartomatous polyps NOS	33 (9%)	6.4%–44%	0.15%
Ganglioneuromas	34 (9%)	3.2%–50%	N/A
Lymphoid polyps	1 (0%)	35%–58%	N/A
Inflammatory and juvenile	28 (7%)	100%^a^	0.1%
Adenomas	55 (15%)	34%–42%	0.69%
Hyperplastic polyps	35 (9%)	33%^a^	17%
Sessile serrated lesions	10 (3%)	N/A	8.2%
Glycogenic acanthosis	6 (2%)	4%–48%	3%–15%
Skin	180 (47%)	14%–100%	
Lipoma	57 (15%)	26.5%–56.7%	1 in 1000
Trichilemmoma	12 (3%)	19%–38%	N/A
Papilloma	25 (7%)	41%–85%	0.84%–12.9%
Acanthosis nigricans	1 (0.2%)	N/A	7%–74%
Fibroma	74 (20%)	0.6%–24%	3%
Skin tag	11 (2%)	16%^a^	46%
Thyroid	118 (31%)	24%–87%	
Benign multinodular hyperplasia	75 (20%)	44.1%–84%	4%
Follicular adenomas	38 (10%)	23%^a^	2%–4.3%
Hashimoto's thyroiditis	5 (1%)	3%–25%	7.5%
Other			
Vascular malformation	99 (26%)	6.4%–35%	4.55%
Lhermitte‐Duclos disease	8 (2%)	3%–13%	N/A
Buccal hamartoma	1 (0.2%)	11%^a^	N/A
Breast	119 (57%)	9%–74%	
Fibrocystic breast disease	66 (31%)	26.4%–52%	50%
Fibromas/fibroadenomas	40 (19%)	32%^a^	25%
Papilloma	13 (6%)	14%^a^	5%–10%
Uterus	49 (23%)	52%	
Fibroid	15 (7%)	5%–26.6%	20%–77%
Hyperplasia	34 (16%)	28%^a^	1.5%–5%

*Note:* Listed are the estimates for PHTS related lesions and for uterine lesions based on frequencies observed in this cohort, PHTS estimates from literature and estimated general population prevalences.

Abbreviation: N/A: not available.

^a^ Only a single study with an available estimate.

### 3.3 Gastrointestinal Lesions

Gastrointestinal lesions were observed in 54% of the total cohort and in 80% of patients above surveillance age (> 40 years): 85% of females and 72% of males. The most frequent group of polyps were hamartomatous polyps (25%, 43 years (IQR 35–50)), consisting of hamartomatous polyps NOS (9%), ganglioneuromas (9%) and inflammatory/juvenile polyps (7%). Lymphoid polyps were present in one patient. Adenomas were observed in 15% (46 years (IQR 38–56)), followed by hyperplastic polyps (9%, 48 years (IQR 45–56)), sessile serrated lesions (3%, 49 years (IQR 46–57)) and glycogenic acanthosis (1.6%, 42 years (IQR 35–52)). The number of polyps per patient per pathology excerpt ranged from 1 to 37 polyps and 1 to 11 for gastrointestinal adenomas. All gastrointestinal lesions were slightly more frequent in females, except glycogenic acanthosis, which was slightly more frequent in males compared to females (3% vs. 0.5%).

### 3.4 Skin Lesions

**FIGURE 2:**
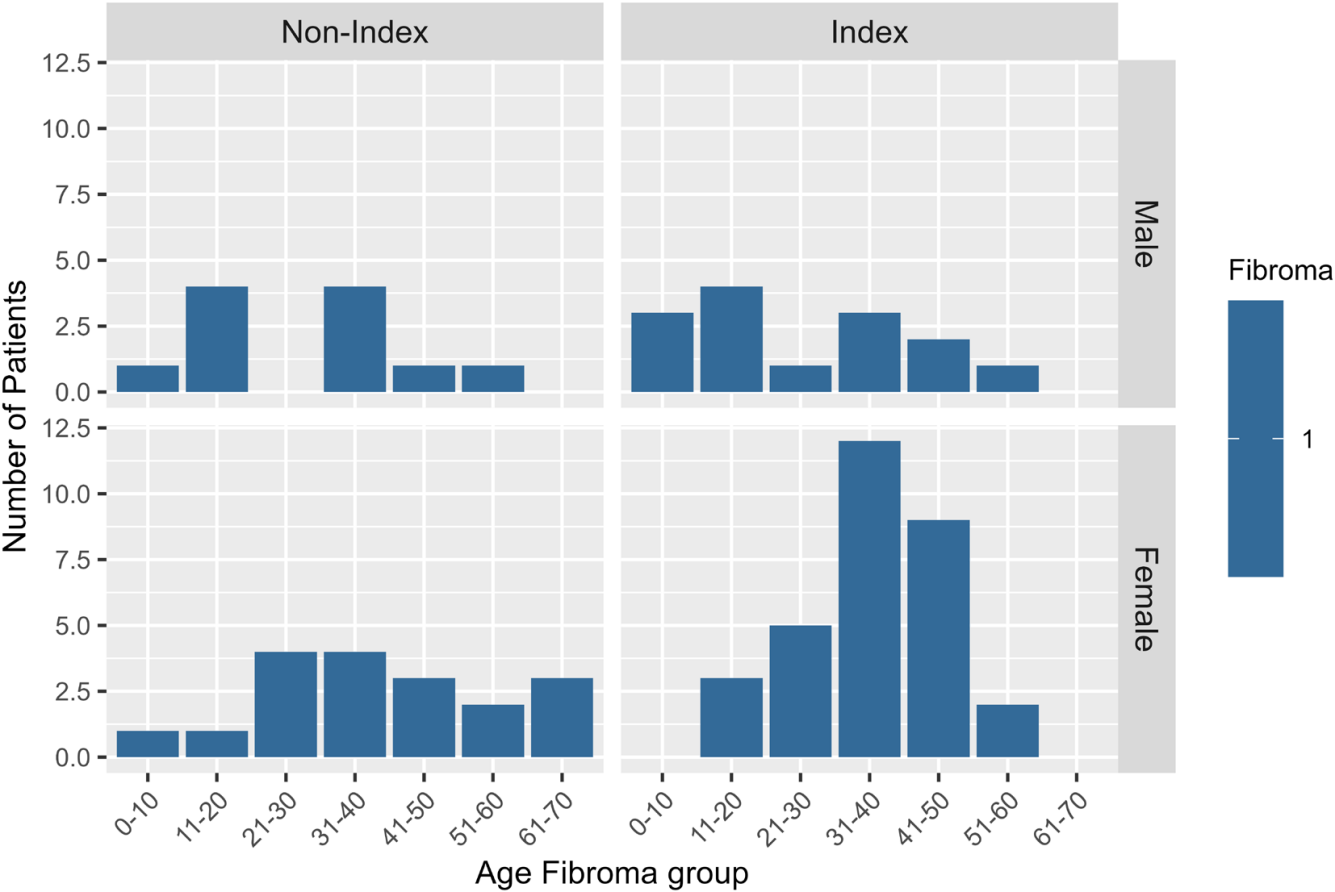
Fibroma age groups by sex and index status. The blue bar indicates number of patients per age group, stratified by sex (horizontally) and index status (vertically) with a pathology‐confirmed fibroma. A total number of 74 patients had one or more pathology‐confirmed fibromas. Reflective of the results in Table 3, fibromas are more frequent in females and especially in index females.

**FIGURE 3:**
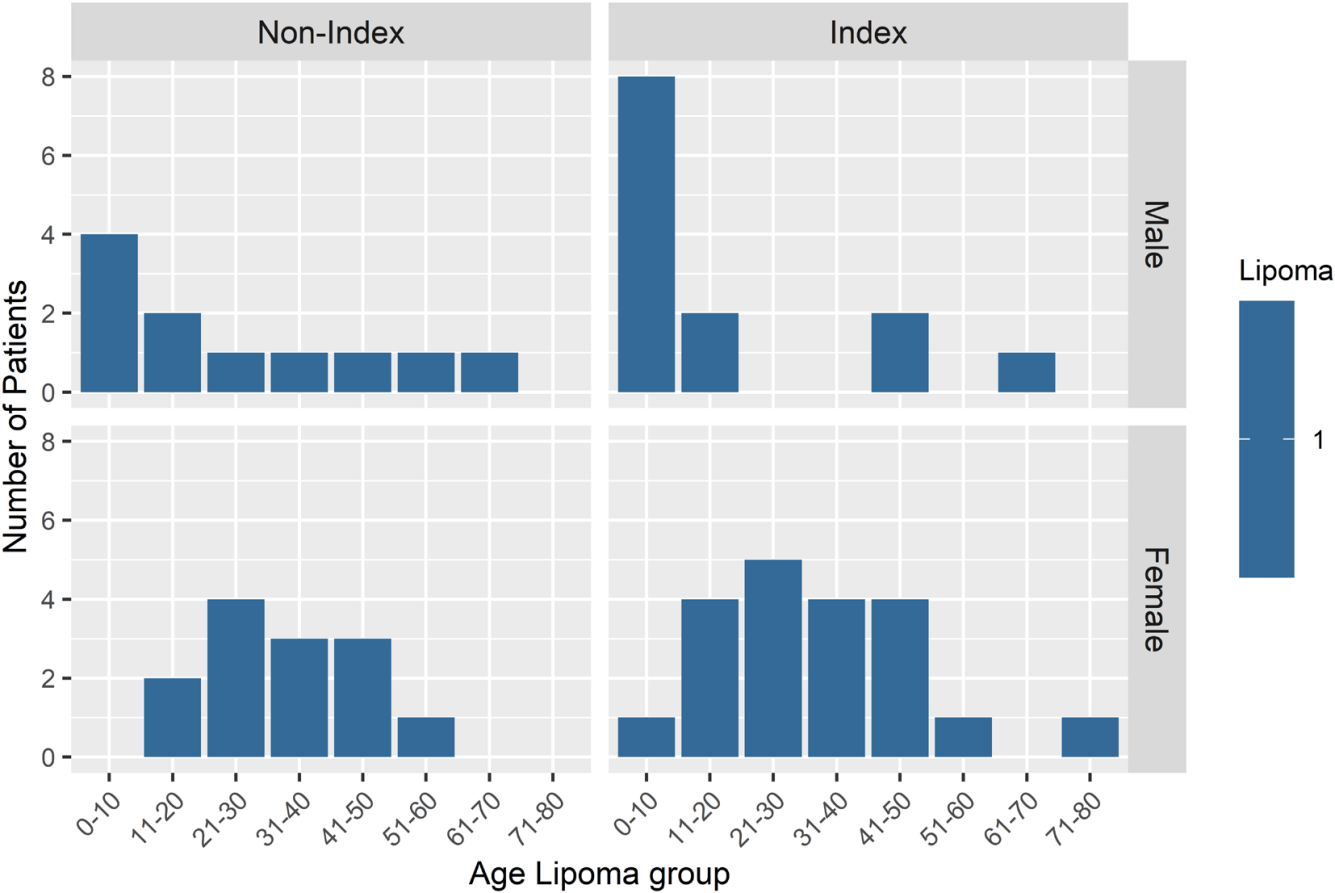
Lipoma age groups by sex and index status. The blue bar indicates number of patients per age group, stratified by sex (horizontally) and index status (vertically) with a pathology‐confirmed lipoma. A total of 57 patients had one or more pathology‐confirmed lipomas. Reflective of the results in Table 3, lipomas are more frequent in females although males were younger at lipoma biopsy/removal.

Benign skin lesions were present in 47% of the PHTS patients and consisted of fibromas (20%, 33 years (IQR 22–42)), lipomas (15%, 24 years (IQR 10–41)), papillomas (7%, 30 years, (IQR 24–38)), trichilemmomas (3%, 35 years (IQR 30–37)), skin tags (2%, 30 years (IQR 19–37)) and one patient with acanthosis nigricans (11 years). Fibromas were more frequent in females compared to males (23% vs. 15%) although males were younger at fibroma excerpt (26 years (IQR 13–34) vs. 36 years (IQR 27–42)). This remained when stratifying for index status (Figure 2). Males were also younger at lipoma excerpt compared to females (9 years (IQR 5–34) vs. 30 years (IQR 22–41)) also when stratifying for index status (Figure 3). Females were younger at trichilemmoma excerpt (31 years (IQR 28–36) vs. 38 years (IQR 38–49)). Non‐index cases were younger at papilloma excerpt compared to index cases (14 years (IQR 8–26) vs. 35 years (28–40)).

### 3.5 Thyroid Lesions

Thyroid lesions were observed in 31% of the total cohort and in 35% of patients above surveillance age (> 11 years): 42% of females and 24% of males. Thyroid lesions consisted of benign multinodular hyperplasia (20%) with a median excerpt age of 38 years (IQR 23–49), follicular adenomas (10%) at a median excerpt age of 35 years (IQR 23–44) and Hashimoto's thyroiditis (1.3%) with a median excerpt age of 41 years (IQR 40–42).

### 3.6 Vascular Malformations

Vascular malformations were present in 26% of patients, consisting of arteriovenous malformation, lymphangiomas, and hemangiomas at a median age of 27 years (IQR 20–35) and were more frequent in females compared to males (34% vs. 16%) although not statistically significant.

### 3.7 Lhermitte‐Duclos Disease

Eight patients (2%, six females) presented with Lhermitte‐Duclos disease at a median age of 36 years (IQR 34–41).

### 3.8 Breast Lesions

Benign breast lesions were observed in 57% of the females and in 76% of females above surveillance age (> 25 years) in this cohort, most frequently as fibrocystic breast disease (31%) at a median age of 38 years (IQR 31–45), fibromas/fibroadenomas (19%) at a median age of 28 years (IQR 19–39) and intraductal papillomas (6%) at a median age of 27 years (IQR 20–39). Non‐index cases trended toward being younger compared to index cases at fibroma/fibroadenoma excerpt (23 years (IQR 17–36) vs. 31 years (IQR 21–43)). No benign breast lesions were observed in males.

### 3.9 Female‐Specific Lesions

In addition to the PHTS‐related lesions, female‐specific benign lesions were also observed with endometrial hyperplasia (16%) at a median age of 46 years (IQR 38–51), uterine polyps (13%), uterine fibroids (7%) at a median age of 41 years (IQR 35–49), endometriosis (5%) and ovarian cysts (5%), cervical polyps (2%) and benign neoplasms of the ovary (3%) being most frequent. Index cases trended toward being younger at uterine fibroid excerpt (41 years (IQR 37–49) vs. 52 years (IQR 46–64)).

### 3.10 Other Observed Lesions

Other non‐PHTS related benign lesions were observed in 32% of patients. The most frequently observed lesions were epidermal cysts (7%), seborrheic keratosis (3%) and ganglion (1%). No statistically significant differences were observed between sexes or by index status. The frequencies of other observed lesions are presented in Supporting Information Table 2.

## 4 Discussion

This large retrospective cohort study, consisting of 379 PHTS patients, assessed the spectrum of PHTS‐related and other observed pathology‐confirmed benign lesion frequencies and excerpt age stratified by sex and index status. The most frequent PHTS‐related lesions were gastrointestinal lesions (53%) followed by skin (47%), thyroid (35%) and vascular lesions (26%). Female‐specific lesions consisted mainly of fibrocystic breast disease (31%), breast fibroadenomas (19%) and intraductal papillomas (6%) were most frequent, followed by endometrial hyperplasia (16%) and uterine fibroids (7%).

Of other benign lesions, uterine polyps (13%), epidermal cysts (7%), endometriosis (5%), ovarian cysts (5%), benign neoplasms of the ovary (3%), seborrheic keratosis (3%) and ganglion (1%) were the most frequent.

The spectrum of observed benign lesions in this study is in line with previous studies and current PHTS diagnostic guidelines and consists of hamartomas involving several body systems and tissue types, reflective of the hamartomatous nature of this syndrome [2, 6, 10]. There were no major significant differences observed between females and males or between index cases and non‐index cases, indicating that the observed lesions seemingly do not have a sex preference and are not due to selection bias.

### 4.1 GI Lesions

Gastrointestinal lesions were observed in 54% of the patients, which is higher than that observed by Hendricks et al. (16%) and Starink et al. (43%) but much less than that observed by Bubien et al. (85%), Drissen et al. (89%) and Pilarski (95%) [1, 10, 12, 13, 14]. The discrepancies observed between the different studies may be reflective of different data types, country guidelines, and/or age of the patients. Hamartomatous polyps (25%) were most frequent, followed by adenomas (15%) which highlights the relevance of hamartomatous polyps as a major criterion of PHTS [1, 6, 10, 13]. Polyps in our cohort were detected mostly above age 40, as many lesions will have been identified upon colon cancer surveillance, which starts at age 40 in the Netherlands [6].

### 4.2 Skin

Approximately half of the patients (47%) presented with one or more skin lesions, which is similar to that observed by Hendricks et al. (38%) and Drissen et al. (14%–78%) but less than that observed by Bubien et al. (98%) and in the review by Pilarski (≤ 100%) [1, 2, 10, 12]. This is partly due to certain lesions, such as oral papillomas, acral keratosis, keratotic pits, trichilemmomas, and acanthosis nigricans, being less likely to get biopsied and are consequently less present or not present in the dataset [15, 16]. Skin lesions overall were more frequent in females compared to males (55% vs. 38%). A probable explanation for this difference could be that females were significantly older than the males in this cohort and had more time to develop these lesions. Additionally, females may be more likely to have skin lesions removed for esthetic reasons, as well as some skin lesions (trichilemmomas and fibromas) being more frequent in females in general [17, 18].

About 15% of the patients had one or more lipomas, which is less than that observed by Drissen et al. (39%), Bubien et al. (48%), Lim and Ngeow (34.6%–56.7%) and Starink et al. (31%) [2, 10, 13, 19]. However, this is still remarkably higher than the incidence of lipomas in the general population, where it is estimated to be present in 1 in 1000 [20]. The patients developed lipomas at a young age (24 (IQR 10–41)) and males were younger at lipoma onset compared to females (9 vs. 30) as well as when stratifying for index status. Moreover, this early onset is further highlighted considering lipomas generally develop between 40 and 60 years of age in the general population [20, 21]. Early‐onset lipomas (< 25 years) may be an indication to consider diagnostic *PTEN* testing.

### 4.3 Thyroid

Approximately 31% of the patients presented with one or more benign thyroid lesions. This is similar to that observed by Tan et al. (35.4%) and Hendricks et al. (24%), but lower than that of Bubien et al. (71%), Starink et al. (68%) and Drissen et al. (84% & 87%), respectively [2, 4, 10, 12, 13, 22]. This is likely due to many thyroid lesions not being biopsied or excised. In the Netherlands, thyroid cancer surveillance is initiated in childhood by physical examination from diagnosis and by yearly ultrasounds from 18 years onwards, with newer studies recommending surveillance start from as young as 12 years old [6, 23].

### 4.4 Vascular

Vascular malformations were present in 26% of patients with a median age of 27 years (IQR 20–35). This is similar to that observed by Bubien et al. (35%) and Drissen et al. (30%) and higher than that by Hendricks et al. (12%) and Starink et al. (18%) [2, 10, 12, 13]. These estimates are still significantly higher than that observed in the general population (5%) which further highlights vascular malformations as an indication of PHTS [24].

### 4.5 Breast Lesions

Benign breast lesions were observed in 57% of the females in this cohort, which is more than that observed by Drissen et al. (35%) [25]. Benign breast lesions in this cohort consisted of fibrocystic breast disease (31%), fibromas/fibroadenomas (19%) and intraductal papillomas (6%). Fibrocystic breast disease (31%) was less frequent in this cohort than that observed by Tan et al. (44.9%) and Starink et al. (52%) [4, 13]. However, fibrocystic breast disease is estimated to affect 50% of women from age 30 onwards in the general population [26]. It is likely that fibrocystic breast disease in this cohort is a reflection of the frequencies in the general population and not specific for PHTS patients.

Fibroadenomas were present in 19% of PHTS females in our study, which is less than that seen by Schrager et al. (32%) and that observed in the general population (25%) [26, 27]. Intraductal papillomas were seen in 6% of PHTS females, which is less than that observed by Schrager et al. (14%) and similar to that observed in the general population (5%–10%) [27]. Benign breast lesions in this cohort were less frequent than that previously described in the literature. However, due to the nature of this data, it may not showcase the full extent of benign breast lesions or their specificity as PHTS‐related lesions.

### 4.6 Female‐Specific Lesions

Benign female‐specific lesions were observed, consisting of endometrial hyperplasia (16%), uterine polyps (13%), uterine fibroids (7%), endometriosis (5%) and ovarian cysts (5%), cervical polyps (2%) and benign neoplasms of the ovary (3%) being most frequent. Endometrial hyperplasia is a precursor lesion of endometrial cancer and is estimated to be present in 1.5%–5% of asymptomatic females in the general population and has been observed in 28% of PHTS females in a previous study [28, 29]. The incidence rates of hyperplasia usually reach their peak when females are in their early 50s to 60s following menopause [28]. However, the estimates of hyperplasia in the general population are uncertain as it rarely gets detected unless it causes abnormal bleeding [28]. As PHTS females have an increased risk of developing endometrial cancer by age 60 of 12%–40%, it is not surprising that hyperplasia is increased in this cohort. The age of hyperplasia in our cohort was much younger (41, IQR 35–49) than that observed in the general population, which is likely reflective of an earlier cancer onset [9, 30]. Additionally, as initiation of endometrial cancer surveillance begins at age 40 in the Netherlands for PHTS patients, this increased rate may indicate that surveillance is effective as hyperplasia is detected before it evolves into endometrial cancer [6]. However, due to the mostly asymptomatic nature of this lesion, it is rarely investigated, and therefore the clinical utility of endometrial hyperplasia for early recognition of PHTS patients is questionable.

Uterine polyps (13%) were the most frequent non‐PHTS‐related female‐specific lesion. In a previous study on endometrial cancer surveillance in PHTS, a single uterine polyp was observed [29]. Uterine polyps were observed in 1.75% of Lynch Syndrome patients undergoing endometrial cancer surveillance due to their increased lifetime endometrial cancer risk (27%–70%) [31]. Furthermore, uterine polyps were observed in 7.8% of patients in a Danish population screening study [32]. Taken together, this indicates that female PHTS patients may develop uterine polyps at an increased rate compared to the general population.

## 5 Strengths

This study consisted of a large cohort of 379 PHTS patients with confirmed likely pathogenic/pathogenic germline *PTEN* variants. The data in this study was extracted from pathology reports from the Dutch nationwide pathology databank, which provides certainty of pathological diagnosis. Stratifying patients by index status and sex allowed us to correct for selection bias and assess whether lesions occur more frequently in one sex. As we had all excerpts for each patient available, we were able to collect and assess frequencies of any other occurring lesions not yet associated with the PHTS phenotype.

## 6 Limitations

Due to the nature of the data, a complete phenotyping of patients was not possible as there was no information on macrocephaly, developmental level, behavioral phenotype, etc. Additionally, not all benign lesions will be sampled or removed, and consequently, the results are an underestimation of the actual presence. Furthermore, some lesions will only be detected upon initiation of cancer surveillance, which is reflected in the similar age of biopsy for all gastrointestinal lesions and endometrial lesions [6]. Consequently, the true age of onset is likely earlier for these lesions.

## 7 Conclusion

The observed lesions in this large cohort study are reflective of current PHTS guidelines and previous literature. Males developed lipomas at a very early age, highlighting the relevance of lipomas as a PHTS‐related lesion. Females developed endometrial hyperplasia at an earlier age compared to the general population and developed uterine polyps more often compared to Lynch syndrome patients as well as the general population. No significant differences were observed between sexes or index cases and non‐index cases, indicating that lesions are PHTS‐specific and not due to selection bias. The results of this study are reflective of the previously observed phenotype and current PHTS diagnostic guidelines. Early‐onset lipomas may be useful for early detection of PHTS patients, and uterine polyps should be considered for inclusion as a PHTS‐related benign lesion.

## Author Contributions

**Ane J. Schei‐Andersen:** conceptualization, data curation, methodology, formal analysis, investigation, writing – original draft, review and editing, visualization. **Janneke H. M. Schuurs‐Hoeijmakers:** writing – review and editing, supervision. **Rachel van der Post:** writing – review and editing. **Arjen R. Mensenkamp:** writing – review and editing. **Jolanda Schieving:** writing – review and editing. **Nicoline Hoogerbrugge:** conceptualization, writing – review and editing, supervision. **Janet R. Vos:** conceptualization, data curation, writing – review and editing, supervision. **PTEN Study Group:** review and editing. The work reported in the paper has been performed by the authors unless clearly specified in the text.

## Ethics Statement

This study (CMO light, dossier number 2019‐5291) was approved by the Medical Ethics Review Committee Oost‐Nederland, based on the criterium that patients consent to (opt‐in) or did not object to (opt‐out) secondary use of health data. The study was also reviewed by the scientific council and privacy committee of Palga.

## Conflicts of Interest

The authors declare no conflicts of interest.

## Supporting information


**Data S1.** Supporting Information.

## Data Availability

The data that support the findings of this study are available on request from the corresponding author. The data are not publicly available due to privacy or ethical restrictions.
