# Supplementary material for: Deep Phenotyping of Pathology‐Confirmed Benign Lesions in PTEN Hamartoma Tumor Syndrome Patients
Source: Clin Genet. 2025 Apr 28;108(4):400–11. doi: 10.1111/cge.14759 (PMC12405060; doi:10.1111/cge.14759)
Supplement: Supplementary file 1 — Data S1. Supporting Information. [file CGE-108-400-s001.docx]

**Supplementary Table 1. Frequency of index patients and non-index patients per benign lesion and excerpt age.**

| **Feature, N (%)** | | | **Total** | **Age, median (IQR)** | **Frequency, *Index*** | | **Index age,**  **median, (IQR)** | | **Frequency,**  ***Non-index*** | | **Non-index age,**  **median, (IQR)** | | **P-value**  **Index status** | | | **P-value Age** |
| --- | --- | --- | --- | --- | --- | --- | --- | --- | --- | --- | --- | --- | --- | --- | --- | --- |
| **GASTROINTESTINAL** | | |  |  |  | |  | |  | |  | |  | | |  |
| *Hamartomatous Polyps* | | | 96 (25%) | 43 (35 - 50) | 54 (24%) | | 42 (35 - 50) | | 42 (25%) | | 43 (34 - 50) | | 0.6661 | | | 0.971 |
| *Hamartomatous polyps NOS* | 33 (9%) | 43 (41 - 47) | 18 (8%) | | 41 (37 - 46) | | 15 (9%) | | 44 (42 - 47) | | 0.5992 | | | 0.3062 |  |  |
| *Ganglioneuromas* | 34 (9%) | 43 (39 - 50) | 20 (9%) | | 41 (36 - 50) | | 14 (8%) | | 44 (41 - 49) | | 0.6784 | | | 0.5615 |  |  |
| *Lymphoid polyps* | 1 (0%) | 46 | 1 (0%) | | 46 | | - | | - | | - | | | - |  |  |
| *Inflammatory & Juvenile* | 28 (7%) | 38 (26 - 44) | 15 (7%) | | 41 (34 - 50) | | 13 (8%) | | 42 (32 - 48) | | 0.9425 | | | 0.8173 |  |  |
| *Adenomas* | | | 55 (15%) | 46 (40 - 58) | 36 (17%) | | 46 (40 - 57) | | 19 (11%) | | 48 (42 - 58) | | 0.7991 | | | 0.3294 |
| *Hyperplastic polyps* | | | 35 (9%) | 48 (45 - 56) | 23 (11%) | | 47 (42 - 54) | | 12 (7%) | | 50 (45 - 57) | | 0.1994 | | | 0.2962 |
| *Sessile serrated lesions* | | | 10 (3%) | 49 (46 - 57) | 5 (2%) | | 50 (48 - 55) | | 5 (3%) | | 47 (44 - 58) | | 0.7466 | | | 0.6905 |
| *Glycogenic acanthosis* | | | 6 (2%) | 42 (35 - 52) | 2 (1%) | | 37 (35 - 38) | | 4 (2%) | | 49 (38 - 59) | | 0.4286 | | | 0.5333 |
| **SKIN** | | |  |  |  | |  | |  | |  | |  | | |  |
| *Lipoma* | | | 57 (15%) | 24 (10 - 41) | 33 (15%) | | 22 (8 - 40) | | 24 (14%) | | 26 (15 - 42) | | 0.6461 | | | 0.3483 |
| *Trichilemmoma* | | | 12 (3%) | 35 (30 - 37) | 6 (3%) | | 34 (31 - 38) | | 6 (4%) | | 36 (30 - 37) | | 0.4725 | | | 0.9358 |
| *Papilloma* | | | 25 (7%) | 30 (24 - 38) | 18 (8%) | | 35 (28 - 40) | | 7 (4%) | | 14 (8 - 26) | | 0.1502 | | | 0.002976 |
| *Fibroma* | | | 74 (20%) | 33 (22 - 42) | 45 (21%) | | 32 (24 -40) | | 29 (17%) | | 34 (22 - 47) | | 0.447 | | | 0.6103 |
| *Skin tag* | | | 11 (3%) | 30 (19 - 37) | 3 (1%) | | 24 (18 - 30) | | 8 (5%) | | 32 (20 - 39) | | 0.0649 | | | 0.497 |
| **THYROID** | | |  |  |  | |  | |  | |  | |  | |  |  |
| *Benign Multinodular Hyperplasia* | | | 75 (20%) | 38 (23 - 49) | 41 (19%) | | 38 (23 - 47) | | 34 (20%) | | 39 (26 - 50) | | 0.5804 | | | 0.5981 |
| *Follicular Adenomas* | | | 38 (10%) | 35 (23 - 44) | 19 (9%) | | 33 (28 - 42) | | 19 (11%) | | 41 (22 - 45) | | 0. 5133 | | | 0.4923 |
| *Hashimoto's thyroiditis* | | | 5 (1%) | 41 (40 - 42) | 2 (1%) | | 42 (41 - 42) | | 3 (2%) | | 40 (28 - 45) | | 0.4286 | | |  |
| **OTHER** | | |  |  |  | |  | |  | |  | |  | | |  |
| *Vascular malformation* | | | 99 (26%) | 27 (20 - 35) | 53 (53%) | | 26 (16 - 37) | | 46 (28%) | | 28 (25 - 33) | | 0.5557 | | | 0.2982 |
| *Lhermitte-Duclos disease* | | | 8 (2%) | 36 (34 - 41) | 6 (3%) | | 35 (33 - 39) | | 2 (1%) | | 44 (40 - 49) | | 0.4743 | | | 0.4286 |
| *Any other abnormality* | | | 121 (32%) | 33 (22 - 42) | 61 (29%) | | 33 (22 - 40) | | 60 (36%) | | 30 (22 - 49) | | 0.1495 | | | 0.5748 |
| **BREAST** | | |  |  |  | |  | |  | |  | |  | | |  |
| *Fibrocystic breast disease* | | | 66 (17%) | 38 (32 - 45) | 37 (17%) | | 39 (34 - 45) | | 29 (17%) | | 34 (32 - 38) | | 0.6085 | | | 0.09662 |
| *Fibromas/ Fibroadenomas* | | | 40 (11%) | 28 (19 - 39) | 18 (8%) | | 31 (21 - 43) | | 22 (13%) | | 23 (17 - 36) | |  | | | 0.05911 |
| *Hamartoma* | | | - |  | - | |  | | - | |  | |  | | |  |
| *Papilloma* | | 13 (3%) | 27 (20 - 39) | | 8 (4%) | | 27 (24 - 41) | | 5 (3%) | | 20 (17 - 33) | | 0.5238 | |  |  |
| **UTERUS** | | |  |  |  | |  | |  | |  | |  | | |  |
| *Fibroid* | | | 15 (4%) | 46 (38 - 51) | 9 (4%) | | 41 (37 - 49) | | 6 (4%) | | 52 (46 - 64) | | 0.5147 | | | 0.09866 |
| *Hyperplasia* | | | 34 (9%) | 41 (35 - 49) | 21 (10%) | | 41 (36 - 49) | | 13 (8%) | | 37 (33 - 50) | | 0.7364 | | | 0.8313 |

**Supplementary Table 2. Frequency all other benign lesions by ICD-10 codes.**

| **ICD-10** | **Total** | **Age,**  **Median (IQR)** | **Female** | **Age,**  **Median (IQR)** | **Male** | **Age,**  **Median (IQR)** | **P-value**  **Sexes** | **P-value Age** |
| --- | --- | --- | --- | --- | --- | --- | --- | --- |
| **A63.0**  *Condyloma* | 1 | 25 | 1 | 25 | 0 | - | 1 |  |
| **B07**  *Verrucosities* | 7 | 51 (38 - 57) | 4 | 46 (40 - 52) | 3 | 58 (45 - 62) | 0.1559 | 0.6286 |
| **B08.1**  *Molluscum contagiosum* | 1 | 36 | 0 | - | 1 | 36 | 1 |  |
| **D11**  *Benign neoplasm of major salivary glands* | 1 | 26 | 1 | 26 | 0 | - | 1 |  |
| **D16.3**  *Short bones of lower limb* | 1 | 10 | 1 | 10 | 0 | - | 1 |  |
| **D21.9**  *Other benign neoplasms of connective and other soft tissue, unspecified* | 2 | 30 (21 - 38) | 2 | 30 (21 - 38) | 0 | - | 1 |  |
| **D23.0**  *Other benign neoplasm of skin of lip* | 1 | 37 | 1 | 37 | 0 | - | 1 |  |
| **D23.3**  *Other benign neoplasm of skin of other unspecified part of face* | 4 | 48 (34 - 53) | 2 | 48 (45 - 50) | 2 | 31 (19 - 43) | 0.1889 | 1 |
| **D23.5**  *Other benign neoplasm of skin of trunk* | 2 | 23 (15 - 30) | 1 | 38 | 1 | 7 | 0.4167 | 1 |
| **D23.7**  *Other benign neoplasm of skin of lower limb, including hip* | 1 | 35 | 1 | 35 | 0 | - | 1 |  |
| **D23.9**  *Other benign neoplasm of skin, unspecified* | 4 | 40 (35 - 45) | 4 | 40 (35 - 45) | 0 | - | 0.5841 |  |
| **D27**  *Benign neoplasm of ovary* | 7 | 21 (17 - 33) | 7 | 21 (17 - 33) | 0 | - | 0.3508 |  |
| **D28.0**  *Benign neoplasm of vulva* | 2 | 39 (38 - 39) | 2 | 39 (38 - 39) | 0 | - | 1 |  |
| **D30.0**  *Benign neoplasm of kidney* | 1 | 70 | 0 | - | 1 | 70 | 1 |  |
| **D32**  *Benign neoplasm of meninges* | 1 | 41 | 0 | - | 1 | 41 | 1 |  |
| **D32.9**  *Benign neoplasm of meninges, unspecified* | 1 | 76 | 0 | - | 1 | 76 | 1 |  |
| **D33.2**  *Benign neoplasm of brain, unspecified* | 1 | 35 | 1 | 35 | 0 | - | 1 |  |
| **D35.1**  *Benign neoplasm of parathyroid gland* | 1 | 74 | 1 | 74 | 0 | - | 1 |  |
| **D36.1**  *Benign neoplasm of peripheral nerves and autonomic nervous system* | 3 | 18 (15 - 20) | 1 | 18 | 2 | 17 (14 - 19) | 0.1376 | 1 |
| **D36.7**  *Benign neoplasm of other specified sites* | 1 | 42 | 1 | 42 | 0 | - | 1 |  |
| **D37.0**  *Neoplasm of uncertain behavior of the lip, oral cavity and pharynx* | 3 | 52 (49 - 54) | 0 | - | 3 | 52 (49 – 54) | 0.008364 |  |
| **D46.9**  *Myelodysplastic syndrome, unspecified* | 1 | 51 | 1 | 51 | 0 | - | 1 |  |
| **D48.7**  *Neoplasm of uncertain behavior of other specified sites* | 3 | 16 (14 - 34) | 2 | 34 (25 - 42) | 1 | 12 | 0.5009 | 1 |
| **D72.9**  *Disorder of white blood cells, unspecified* | 1 | 33 | 0 | - | 1 | 33 | 1 |  |
| **D76.3**  *Other histiocytosis syndromes* | 1 | 24 | 1 | 24 | 0 | - | 1 |  |
| **E78.5**  *Hyperlipidaemia, unspecified* | 1 | 54 | 1 | 54 | 0 | - | 1 |  |
| **G56.1**  *Other lesions of median nerve* | 1 | 11 | 1 | 11 | 0 | - | 1 |  |
| **G57.6**  *Lesion of plantar nerve* | 1 | 54 | 1 | 54 | 0 | - | 1 |  |
| **G72.9**  *Myopathy, unspecified* | 1 | 1 | 1 | 1 | 0 | - | 1 |  |
| **H02.6**  *Xanthelasma of eyelid* | 1 | 48 | 1 | 48 | 0 | - | 1 |  |
| **I62.0**  *Nontraumatic subdural haemorrhage* | 1 | 67 | 1 | 67 | 0 | - | 1 |  |
| **I82.8**  *Embolism and thrombosis of other specified veins* | 1 | 37 | 1 | 37 | 0 | - | 1 |  |
| **I82.9**  *Embolism and thrombosis of other unspecified vein* | 1 | 13 | 1 | 13 | 0 | - | 1 |  |
| **J33.0**  *Polyp of nasal cavity* | 3 | 35 (33 - 43) | 0 | - | 3 | 35 (33 - 43) | 0.008364 |  |
| **J33.8**  *Polyp of sinus* | 2 | 28 (24 – 32) | 2 | 28 (24 – 32) | 0 | - | 1 |  |
| **J33.9**  *Nasal polyp, unspecified* | 2 | 49 (48 – 49) | 1 | 49 | 1 | 48 | 0.3702 | 1 |
| **J35.2**  *Hypertrophy of adenoids* | 1 | 4 | 1 | 4 | 0 | - | 1 |  |
| **J35.9**  *Chronic disease of tonsils and adenoids, unspecified* | 1 | 5 | 0 | - | 1 | 5 | 0.2353 |  |
| **J38.1**  *Polyp of vocal cord and larynx* | 1 | 25 | 0 | - | 1 | 25 | 0.2353 |  |
| **J38.7**  *Other diseases of larynx* | 1 | 52 | 0 | - | 1 | 52 | 0.2353 |  |
| **K06.1**  *Gingival enlargement* | 1 | 25 | 1 | 25 | 0 | - | 1 |  |
| **K09.9**  *Cyst of oral region, unspecified* | 1 | 41 | 1 | 41 | 0 | - | 1 |  |
| **K11.8**  *Other diseases of salivary glands* | 1 | 67 | 0 | - | 1 | 67 | 0.2353 |  |
| **K13.6**  *Irritative hyperplasia of oral mucosa* | 2 | 32 (31 – 32) | 1 | 33 | 1 | 30 | 0.3702 | 1 |
| **K13.7**  *Other and unspecified lesions of oral mucosa* | 2 | 56 (46 - 65) | 2 | 56 (46 - 65) | 0 | - | 1 |  |
| **K21.0**  *Gastro-oesophageal reflux disease with oesophagitis* | 1 | 39 | 1 | 39 | 0 | - | 0.206 |  |
| **K31.8**  *Other specified diseases of stomach and duodenum* | 1 | 34 | 0 | - | 1 | 34 | 1 |  |
| **K31.9**  *Disease of stomach and duodenum, unspecified* | 1 | 22 | 1 | 22 | 0 | - | 1 |  |
| **K35**  *Acute appendicitis* | 1 | 21 | 1 | 21 | 0 | - | 1 |  |
| **K38.9**  *Disease of appendix, unspecified* | 1 | 27 | 1 | 27 | 0 | - | 0.206 |  |
| **K52.9**  *Noninfective gastroenteritis and colitis, unspecified* | 1 | 20 | 1 | 20 | 0 | - | 1 |  |
| **K62.3**  *Rectal prolaps* | 1 | 42 | 1 | 42 | 0 | - | 1 |  |
| **K62.8**  *Other specified diseases of anus and rectum* | 1 | 66 | 1 | 66 | 0 | - | 1 |  |
| **K66**  *Other disorders of peritoneum* | 1 | 41 | 1 | 41 | 0 | - | 1 |  |
| **K76.0**  *Fatty liver, not elsewhere classified* | 1 | 72 | 1 | 72 | 0 | - | 1 |  |
| **K80**  *Cholelithiasis* | 1 | 72 | 1 | 72 | 0 | - | 1 |  |
| **K80.1**  *Calculus of gallbladder with other cholecystitis* | 2 | 49 (48 – 50) | 2 | 49 (48 – 50) | 0 | - | 1 |  |
| **K82.8**  *Other specified diseases of gallbladder* | 1 | 52 | 0 | 52 | 1 | - | 1 |  |
| **L05.0**  *Pilonidal cyst with abscess* | 2 | 21 (20 – 21) | 0 | - | 2 | 21 (20 – 21) | 0.04182 |  |
| **L11.0**  *Acquired keratosis follicularis* | 1 | 36 | 1 | 36 | 0 | - | 1 |  |
| **L28.1**  *Prurigo nodularis* | 2 | 37 (33 - 40) | 0 | - | 2 | 37 (33 - 40) | 0.04182 |  |
| **L43**  *Lichen planus* | 1 | 37 | 1 | 37 | 0 | - | 1 |  |
| **L57.0**  *Actinic keratosis* | 3 | 68 (67 - 68) | 0 | - | 3 | 68 (67 – 68) | 0.008364 |  |
| **L66.4**  *Folliculitis ulerythematosa reticulata* | 1 | 38 | 0 | - | 1 | 38 | 1 |  |
| **L72**  *Follicular cysts of skin and subcutaneous tissue* | 1 | 66 | 1 | 66 | 0 | - | 1 |  |
| **L72.0**  *Epidermal cyst* | 25 | 33 (27 - 50) | 21 | 35 (28 - 50) | 4 | 29 (19 - 42) | 0.795 | 0.5775 |
| **L72.2**  *Steatocystoma multiplex* | 1 | 73 | 1 | 73 | 0 | - | 1 |  |
| **L73.2**  *Hidradenitis suppurativa* | 1 | 42 | 0 | - | 1 | 42 | 0.2353 |  |
| **L73.8**  *Other specified follicular disorders* | 3 | 32 (28 – 37) | 3 | 32 (28 – 37) | 0 | - | 1 |  |
| **L81.4**  *Other melanin hyperpigmentation* | 1 | 43 | 1 | 43 | 0 | - | 1 |  |
| **L82**  *Seborroeic keratosis* | 12 | 47 (29 - 65) | 5 | 29 (23 - 34) | 7 | 64 (56 - 66) | 0.003755 | 0.0228 |
| **L85.8**  *Other specified epidermal thickening* | 4 | 51 (43 – 59) | 2 | 41 (35 – 47) | 2 | 62 (55 – 69) | 0.1889 | 0.6667 |
| **L85.9**  *Epidermal thickening, unspecified* | 7 | 49 (32 - 52) | 5 | 49 (39 - 52) | 2 | 50 (37 - 62) | 0.6358 | 0.6667 |
| **L90.5**  *Scar conditions and fibrosis of skin* | 3 | 31 (31 – 40) | 1 | 48 | 2 | 31 | 0.1087 | 0.4795 |
| **L91.0**  *Hypertrophic scar* | 1 | 26 | 1 | 26 | 0 | - | 1 |  |
| **L92.9**  *Granolumatous disorder of skin and subcutaneous tissue, unspecified* | 1 | 33 | 1 | 33 | 0 | - | 1 |  |
| **L98.0**  *Pyogenic granuloma* | 2 | 31 (29 - 32) | 1 | 27 | 1 | 34 | 0.4167 | 1 |
| **L98.9**  *Disorder of skin and subcutaneous tissue, unspecified* | 1 | 34 | 1 | 34 | 0 | - | 1 |  |
| **M06.9**  *Rheumatoid arthritis, unspecified* | 1 | 47 | 1 | 47 | 0 | - | 1 |  |
| **M35.0**  *Sicca syndrome* | 1 | 28 | 1 | 38 | 0 | - | 1 |  |
| **M65**  *Synovitis and tenosyvitis* | 1 | 45 | 1 | 45 | 0 | - | 1 |  |
| **M67.4**  *Ganglion* | 5 | 28 (21 - 49) | 4 | 39 (25 - 49) | 1 | 21 | 1 | 0.8 |
| **M71.1**  *Other infective bursitis* | 1 | 22 | 1 | 22 | 0 | - | 1 |  |
| **M79.3**  *Panniculitis, unspecified* | 1 | 25 | 1 | 25 | 0 | - | 1 |  |
| **M86.9**  *Osteomyelitis, unspecified* | 1 | 15 | 0 | - | 1 | 15 | 0.2353 |  |
| **M89.2**  *Other disorders of bone development* | 1 | 49 | 1 | 49 | 0 | - | 1 |  |
| **N60.0**  *Solitary cyst of breast* | 3 | 37 (36 – 38) | 3 | 37 (36 – 38) | 0 | - | 1 |  |
| **N60.4**  *Mammary duct ectasia* | 1 | 39 | 1 | 39 | 0 | - | 1 |  |
| **N60.8**  *Other benign mammary dysplasias* | 1 | 33 | 1 | 33 | 0 | - | 1 |  |
| **N61**  *Inflammatory disorders of breast* | 1 | 31 | 1 | 31 | 0 | - | 1 |  |
| **N64.1**  *Fat necrosis of breast* | 2 | 32 (30 – 35) | 2 | 32 (30 – 35) | 0 | - | 1 |  |
| **N64.9**  *Disorders of breast, unspecified* | 1 | 46 | 1 | 46 | 0 | - | 1 |  |
| **N80**  *Endometriosis* | 9 | 43 (29 - 49) | 9 | 43 (29 - 49) | 0 | - | 0.2112 |  |
| **N80.1**  *Endometriosis of ovary* | 1 | 39 | 1 | 39 | 0 | - | 1 |  |
| **N83.0**  *Follicular cyst of ovary* | 2 | 40 (38 – 41) | 2 | 40 (38 – 41) | 0 | - | 1 |  |
| **N83.1**  *Corpus luteum cyst* | 1 | 40 | 1 | 40 | 0 | - | 1 |  |
| **N83.2**  *Other and unspecified ovarian cysts* | 10 | 44 (41 - 52) | 10 | 44 (41 - 52) | 0 | - | 0.2228 |  |
| **N84.0**  *Polyp of corpus uteri* | 27 | 43 (37 - 49) | 27 | 43 (37 - 49) | 0 | - | 0.002011 |  |
| **N84.1**  *Polyp of cervix uteri* | 4 | 45 (40 - 46) | 4 | 45 (40 - 46) | 0 | - | 0.5841 |  |
| **N87.9**  *Dysplasia of cervix uteri, unspecified* | 2 | 41 (38 – 43) | 2 | 41 (38 – 43) | 0 | - | 1 |  |
| **N90.7**  *Vulvar cyst* | 1 | 33 | 1 | 33 | 0 | - | 1 |  |
| **O71.7**  *Obstetric haematoma of pelvis* | 1 | 42 | 1 | 42 | 0 | - | 1 |  |
| **Q33.9**  *Congenital malformation of lung, unspecified* | 1 | 0 | 1 | 0 | 0 | - | 1 |  |
| **Q80.0**  *Ichthyosis vulgaris* | 1 | 33 | 1 | 33 | 0 | - | 1 |  |
| **Q82.8**  *Mastocytosis* | 2 | 33 (22 - 44) | 1 | 11 | 1 | 55 | 0.4167 | 1 |
| **Q89.2**  *Congenital malformation of other endocrine glands* | 1 | 35 | 1 | 35 | 0 | - | 1 |  |
| **R23.8**  *Other and unspecified skin changes* | 1 | 51 | 1 | 51 | 0 | - | 0.206 |  |
| **R59.0**  *Localized enlaged lymph nodes* | 3 | 25 (23 - 34) | 3 | 25 (23 - 34) | 0 | - | 0.5009 |  |
| **R59.9**  *Enlarged lymph nodes, unspecified* | 4 | 27 (25 - 32) | 3 | 48 | 1 | 22 | 1 | 1 |
| **R87**  *Abnormal findings in specimens from female genital organs* | 1 | 70 | 1 | 70 | 0 | - | 1 |  |
| **S64.4**  *Injury of digital nerve of other finger* | 1 | 31 | 1 | 31 | 0 | - | 1 |  |
